# Supplementary material for: Cryo-EM structures of the Spo11 core complex bound to DNA
Source: Nat Struct Mol Biol. 2024 Sep 20;32(1):113–24. doi: 10.1038/s41594-024-01382-8 (PMC11746154; doi:10.1038/s41594-024-01382-8)
Supplement: Supplementary file 1 — Supplementary Tables 1 and 2. [file 41594_2024_1382_MOESM1_ESM.pdf]

nature structural & molecular biology

Supplementary information https://doi.org/10.1038/s41594-024-01382-8

Cryo-EM structures of the Spo11 core complex bound to DNA

In the format provided by the authors and unedited

Supplementary Table 1 | Yeast strains

Strain	Genotype
SKY661	*MATa, ho::LYS2, lys2, leu2::hisG, trp1::hisG, ndt80::kanMX, LexA(op)-LacZ::URA3*
SKY662	*MATα, ho::LYS2, lys2, leu2::hisG, trp1::hisG, ndt80::kanMX, LexA(op)-LacZ::URA3*
SKY1311	*MATα, ho::LYS2, lys2, ura3, leu2::hisG, trp1::hisG, arg4-nsp, rec102Δ::URA3*
SKY1312	*MATa, ho::LYS2, lys2, ura3, leu2::hisG, trp1::hisG, arg4-bgl, rec102Δ::URA3*
SKY969	*MATα, ho::LYS2, lys2, leu2::hisG, trp1::hisG, arg4-bgl, spo11Δ::hisG-URA3-hisG*
SKY970	*MATa, ho::LYS2, lys2, leu2::hisG, trp1::hisG, arg4-nsp, spo11Δ::hisG-URA3-hisG*
SKY7404	*MATa, ho::LYS2, lys2, leu2::hisG, trp1::hisG, arg4-bgl, rec104Δ::KanMX*
SKY7405	*MATα,ho::LYS2, lys2, leu2::hisG, trp1::hisG, arg4-nsp, rec104Δ::KanMX*

Supplementary Table 2 | Plasmids

Plasmid	Description	Reference
pCCB586	*SPO11* from *S. cerevisiae* (SK1 strain) in pFastBac1	14
pCCB587	*SKI8* from *S. cerevisiae* (SK1 strain) in pFastBac1	14
pCCB588	*REC102* from *S. cerevisiae* (SK1 strain) in pFastBac1	14
pCCB589	*REC104* from *S. cerevisiae* (SK1 strain) in pFastBac1	14
pSK275	LexA empty Y2H vector	22
pSK276	Gal4AD empty Y2H vector	22
pSK282	LexA-Rec102 Y2H vector (pCA1-Rec102)	23
pSK293	Rec104-LexA Y2H vector	22
pSK305	Gal4AD-Spo11 Y2H vector	22
pSK310	Gal4AD-Rec104 Y2H vector	22
pZZy1	pSK305 with *spo11-L53A*	This study
pZZy2	pSK305 with *spo11-L60A*	This study
pZZy3	pSK305 with *spo11-F91A*	This study
pZZy4	pSK305 with *spo11-L105A*	This study
pZZy5	pSK305 with *spo11-L112A*	This study
pZZy6	pSK305 with *spo11-L93A*	This study
pZZy7	pSK305 with *spo11-N94A*	This study
pZZy8	pSK305 with *spo11-G95A*	This study
pZZy9	pSK305 with *spo11-L98A*	This study
pZZy10	pSK305 with *spo11-F103A*	This study
pZZy11	pSK305 with *spo11-L3A*	This study
pZZy12	pSK305 with *spo11-R6A*	This study
pZZy13	pSK305 with *spo11-R7A*	This study
pZZy14	pSK305 with *spo11-R7D*	This study
pZZy15	pSK305 with *spo11-L16A*	This study
pZZy16	pSK305 with *spo11-V17A*	This study
pZZy17	pSK305 with *spo11-L20A*	This study
pZZy18	pSK282 with *rec102-L207A*	This study
pZZy19	pSK282 with *rec102-I195A*	This study
pZZy20	pSK282 with *rec102-L198A*	This study
pZZy21	pSK282 with *rec102-R199A*	This study

pZZy22	pSK282 with *rec102-W202A*	This study
pZZy23	pSK282 with *rec102-S233A*	This study
pZZy24	pSK282 with *rec102-I249A*	This study
pZZy25	pSK282 with *rec102-L8A*	This study
pZZy26	pSK282 with *rec102-V10A*	This study
pZZy27	pSK282 with *rec102-L179A*	This study
pZZy28	pSK282 with *rec102-S182A*	This study
pZZy29	pSK282 with *rec102-F11A*	This study
pZZy30	pSK282 with *rec102-F156A*	This study
pZZy31	pSK310 with *rec104-I11A*	This study
pZZy32	pSK310 with *rec104-T12A*	This study
pZZy33	pSK310 with *rec104-F17A*	This study
pZZy34	pSK310 with *rec104-L18A*	This study
pZZy35	pSK310 with *rec104-Y21A*	This study
pZZy36	pSK310 with *rec104-F31A*	This study
pZZy37	pSK310 with *rec104-L33A*	This study
pZZy38	pSK310 with *rec104-V50A*	This study
pZZy39	pSK310 with *rec104-I53A*	This study
pZZy40	pSK293 with *rec104-D16A*	This study
pZZy41	pSK293 with *rec104-D16R*	This study
pZZy42	pSK293 with *rec104-Y21A*	This study
pZZy43	pSK305 with *spo11-R13A*	This study
pZZy44	pSK305 with *spo11-K8A*	This study
pZZy45	pSK282 with *rec102-Q183A*	This study
pZZy46	pSK305 with *spo11-Q012A*	This study
pZZy47	pSK282 with *rec102-Q239A*	This study
pZZy48	pSK293 with *rec104-F22A*	This study
pZZy49	pSK293 with *rec104-Q20A*	This study
pZZy50	pSK310 with *rec104-K10A*	This study
