# Supplementary material for: Cryo-EM structures of the Spo11 core complex bound to DNA
Source: Nat Struct Mol Biol. 2024 Sep 20;32(1):113–24. doi: 10.1038/s41594-024-01382-8 (PMC11746154; doi:10.1038/s41594-024-01382-8)
Supplement: Supplementary file 2 — Reporting Summary [file 41594_2024_1382_MOESM2_ESM.pdf]

Corresponding author(s): Scott Keeney and Dinshaw Patel

Last updated by author(s): 6/24/2024

Reporting Summary

Nature Portfolio wishes to improve the reproducibility of the work that we publish. This form provides structure for consistency and transparency in reporting. For further information on Nature Portfolio policies, see our Editorial Policies and the Editorial Policy Checklist.

Statistics

For all statistical analyses, confirm that the following items are present in the figure legend, table legend, main text, or Methods section.

n/a	Confirmed	
☐	☒	The exact sample size (*n*) for each experimental group/condition, given as a discrete number and unit of measurement
☐	☒	A statement on whether measurements were taken from distinct samples or whether the same sample was measured repeatedly
☒	☐	The statistical test(s) used AND whether they are one- or two-sided<br>*Only common tests should be described solely by name; describe more complex techniques in the Methods section.*
☒	☐	A description of all covariates tested
☒	☐	A description of any assumptions or corrections, such as tests of normality and adjustment for multiple comparisons
☐	☒	A full description of the statistical parameters including central tendency (e.g. means) or other basic estimates (e.g. regression coefficient) AND variation (e.g. standard deviation) or associated estimates of uncertainty (e.g. confidence intervals)
☒	☐	For null hypothesis testing, the test statistic (e.g. *F*, *t*, *r*) with confidence intervals, effect sizes, degrees of freedom and *P* value noted<br>*Give P values as exact values whenever suitable.*
☒	☐	For Bayesian analysis, information on the choice of priors and Markov chain Monte Carlo settings
☒	☐	For hierarchical and complex designs, identification of the appropriate level for tests and full reporting of outcomes
☒	☐	Estimates of effect sizes (e.g. Cohen's *d*, Pearson's *r*), indicating how they were calculated

Our web collection on statistics for biologists contains articles on many of the points above.

Software and code

Policy information about availability of computer code

Data collection	Cryo-EM images were collected using SerialEM 3.9.0 (http://bio3d.colorado.edu/SerialEM/)
Data analysis	The following publicly available software was used: Relion v3.0.8 (https://relion.readthedocs.io/en/release-3.1/SPA_tutorial/index.html), Cryosparc v4.2.1 (https://cryosparc.com/), Phenix v1.20.1-4487 (https://phenix-online.org/), Coot 0.8.9.2 (https://www2.mrc-lmb.cam.ac.uk/personal/pemsley/coot/), PyMol v2.5.3 (https://pymol.org/2/), Chimera v1.16 (https://www.cgl.ucsf.edu/chimera/), ChimeraX v. 1.6.1 (https://www.cgl.ucsf.edu/chimerax/), GraphPad Prism v. 10.2.3 (https://www.graphpad.com/scientific-software/prism/www.graphpad.com/scientific-software/prism/), MotionCor2 (ref. 47), CTFFIND-4 (ref. 48), Colabfold (ref. 52), COBALT (ref. 45), NCBI BLAST (https://blast.ncbi.nlm.nih.gov/Blast.cgi).

For manuscripts utilizing custom algorithms or software that are central to the research but not yet described in published literature, software must be made available to editors and reviewers. We strongly encourage code deposition in a community repository (e.g. GitHub). See the Nature Portfolio guidelines for submitting code & software for further information.

Data

Policy information about availability of data

All manuscripts must include a data availability statement. This statement should provide the following information, where applicable:

- Accession codes, unique identifiers, or web links for publicly available datasets
- A description of any restrictions on data availability
- For clinical datasets or third party data, please ensure that the statement adheres to our policy

The atomic coordinates and cryo-EM density maps for the hairpin DNA bound to Spo11 core complex (PDB: 8URU (https://www.rcsb.org/structure/8URU); EMD: EMD-42501 (https://www.ebi.ac.uk/emdb/EMD-42501) and for gapped DNA bound to Spo11 core complex (PDB: 8URQ (https://www.rcsb.org/structure/8URQ); EMD: EMD-42497(https://www.ebi.ac.uk/emdb/EMD-42497) have been deposited in the Research Collaboratory for Structural Bioinformatics Protein Data Bank and Electron Microscopy Data Bank, respectively. We used the following published atomic coordinate accessions: PDB 2ZBK, 2Q2E,1D3Y,1S4U; AlphaFold database (https://alphafold.ebi.ac.uk/) AF-Q02721-F1-model_v1, AF-P33323-F1-model-v1, AF-P23179-F1-model_v1.

Research involving human participants, their data, or biological material

Policy information about studies with human participants or human data. See also policy information about sex, gender (identity/presentation), and sexual orientation and race, ethnicity and racism.

Reporting on sex and gender	N/A
Reporting on race, ethnicity, or other socially relevant groupings	N/A
Population characteristics	N/A
Recruitment	N/A
Ethics oversight	N/A

Note that full information on the approval of the study protocol must also be provided in the manuscript.

Field-specific reporting

Please select the one below that is the best fit for your research. If you are not sure, read the appropriate sections before making your selection.

☒ Life sciences ☐ Behavioural & social sciences ☐ Ecological, evolutionary & environmental sciences

For a reference copy of the document with all sections, see nature.com/documents/nr-reporting-summary-flat.pdf

Life sciences study design

All studies must disclose on these points even when the disclosure is negative.

Sample size	Cryo-EM: No statistical method was used to predetermine the sample size. The number of particles used in structural determination was not pre-determined. Sample size was arbitrarily selected based on the time required to collect data. The sample sizes are sufficient because many independently recorded images were acquired as part of cryo-EM data collection. The particle numbers were sufficient based on the multiple rounds of classification and the quality of the cryo-EM maps. For DNA binding assays, Y2H assays, and heteroallele recombination assays, sample sizes (three independent replicates) were performed in keeping with standard practice in the field for these assays. Immunoblotting was performed at least twice for each sample (this analysis was not quantitative, so additional replicates were not necessary).
Data exclusions	Cryo-EM: Cryo-EM images with bad ice or contamination were removed. Regarding the particle selection, 2D and 3D classification were used and criterion is based on the quality of resulting 2D class average and 3D maps. No data were excluded from DNA binding, heteroallele recombination assays and yeast two-hybrid assays.
Replication	Cryo-EM: At least three rounds of structural refinement have been performed and all resulted in same density maps.<br>DNA binding assays, heteroallele recombination assays and yeast two-hybrid assays were conducted on three independent replicates with similar results. The protein purification was performed more than six times with similar results. Immunoblots were repeated at least twice with similar results. All attempts at replication were successful.
Randomization	Cryo-EM: Particles were randomized following extraction to avoid bias during particle classification. All other experiments involved comparison of mutants to matched wild-type controls, so randomization is neither necessary nor appropriate.
Blinding	No blinding was used for analysis of structural data. All other experiments involved comparison of isogenic control (wild type) and mutant yeast strains. It is not standard practice in the field to use blinding for these assays. Moreover, blinding is not necessary with this experimental design because meaningful effect sizes are larger than any likely effects of operator bias.

Reporting for specific materials, systems and methods

We require information from authors about some types of materials, experimental systems and methods used in many studies. Here, indicate whether each material, system or method listed is relevant to your study. If you are not sure if a list item applies to your research, read the appropriate section before selecting a response.

Materials & experimental systems

n/a	Involved in the study
☐	☒ Antibodies
☐	☒ Eukaryotic cell lines
☒	☐ Palaeontology and archaeology
☒	☐ Animals and other organisms
☒	☐ Clinical data
☒	☐ Dual use research of concern
☒	☐ Plants

Methods

n/a	Involved in the study
☒	☐ ChIP-seq
☒	☐ Flow cytometry
☒	☐ MRI-based neuroimaging

Antibodies

Antibodies used	Anti-Flag M2 affinity resin (Sigma Aldrich, catalog no. A2220); anti-LexA (Sigma Aldrich, catalog no. 06-719); anti-HA (Roche, catalog no. 12013819001); anti-alpha-tubulin (Invitrogen, catalog no. MA1-80017 and Santa Cruz, catalog no. sc-53030); donkey anti-rabbit IgG (Invitrogen, catalog no. A-21206); donkey anti-rat IgG (Invitrogen, catalog no. A-21209).
Validation	The flag epitope was added to the expression construct to produce recombinant Spo11 core complexes in insect cells. Specificity was confirmed by successful purification of Spo11 complexes. The HA epitope is part of the Gal4 activating domain fusion construct. Specificity of HA and LexA antibodies for immunoblotting was validated using control strains that did not express the fusion protein of interest. Tubulin antibody specificity has been established for many species in many applications (summarized at https://www.thermofisher.com/antibody/product/alpha-Tubulin-Antibody-clone-YL1-2-Monoclonal/MA1-80017 and https://www.scbt.com/p/alpha-tubulin-antibody-yol1-34) and confirmed for our experiments using immunoblotting of S. cerevisiae extracts by detection of a band of the appropriate molecular weight.

Eukaryotic cell lines

Policy information about cell lines and Sex and Gender in Research

Cell line source(s)	Yeast strains were generated in the Keeney laboratory.<br>Gibco Spodoptera Frugiperda Sf9 cells for expression of recombinant proteins were from Thermo Fisher (catalog #11496015).
Authentication	Yeast strains were verified by PCR and/or Southern blotting. Mutant protein constructs were confirmed by sequencing.<br>Sf9 description of certification analyses is available at https://www.thermofisher.com/order/catalog/product/11496015#/11496015 "Each lot of Gibco™ Sf9 cells is tested for cell growth and viability post-recovery from cryopreservation. In addition, the Master Seed Bank has been tested for contamination of bacteria, yeast, mycoplasma and virus and has been characterized by isozyme and karyotype analysis." No further authentication was performed in this study.
Mycoplasma contamination	Not applicable for yeast strains. Mycoplasma testing of Sf9 cells is referenced at https://www.thermofisher.com/order/catalog/product/11496015#/11496015. No further mycoplasma testing was done for this study.
Commonly misidentified lines<br>(See ICLAC register)	No commonly misidentified cell lines were used in this study (yeast strains and Sf9 cells only)

Plants

Seed stocks	N/A
Novel plant genotypes	N/A
Authentication	N/A
