# Supplementary figures and images for: Cryo-EM structures of the Spo11 core complex bound to DNA
Source: Nat Struct Mol Biol. 2024 Sep 20;32(1):113–24. doi: 10.1038/s41594-024-01382-8 (PMC11746154; doi:10.1038/s41594-024-01382-8)

Adjusted images

Adjusted images

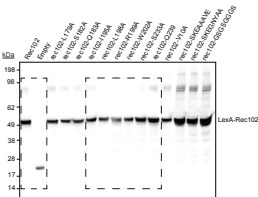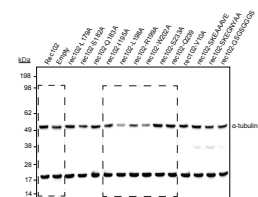

Supplement: Supplementary file 4 — Uncropped immunoblot images. [file 41594_2024_1382_MOESM4_ESM.pdf]

Extended Data Fig. 1 a

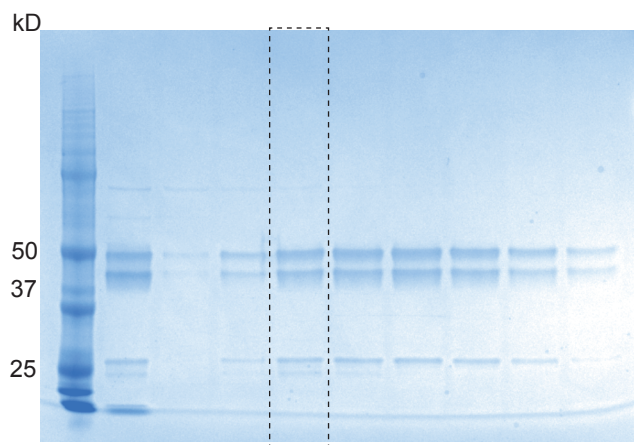

Extended Data Fig. 1 b

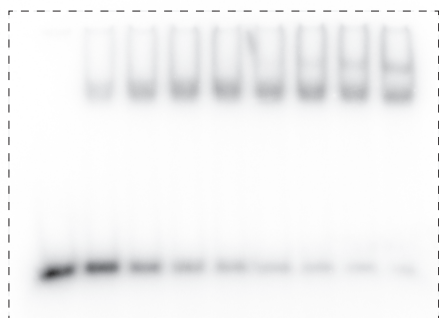

Extended Data Fig. 1 c

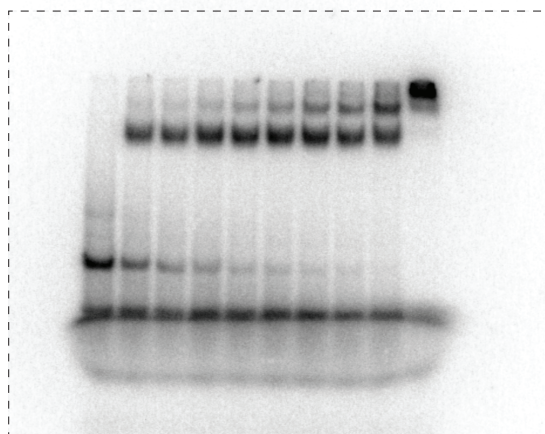

Adjusted images

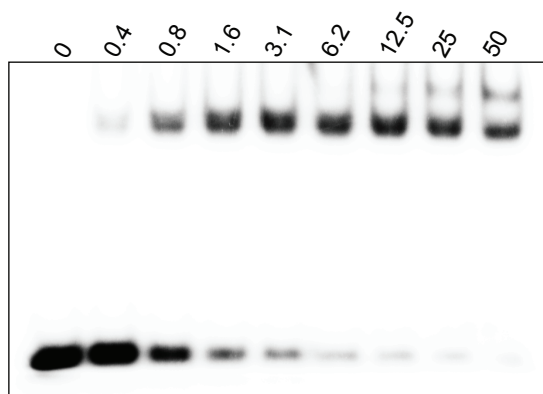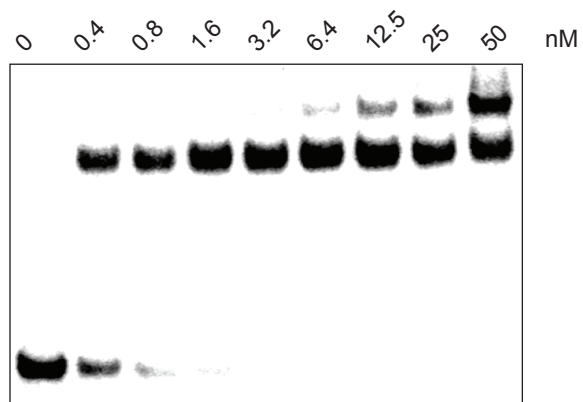

Supplement: Supplementary file 6 — Uncropped gels. [file 41594_2024_1382_MOESM6_ESM.pdf]

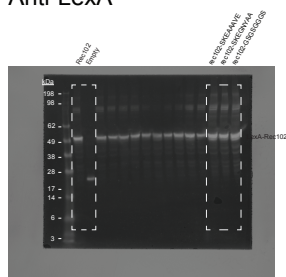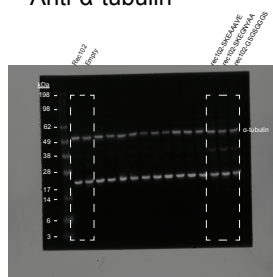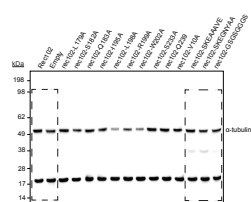

Supplement: Supplementary file 8 — Unprocessed immunoblots. [file 41594_2024_1382_MOESM8_ESM.pdf]

Extended Data Fig. 8 c

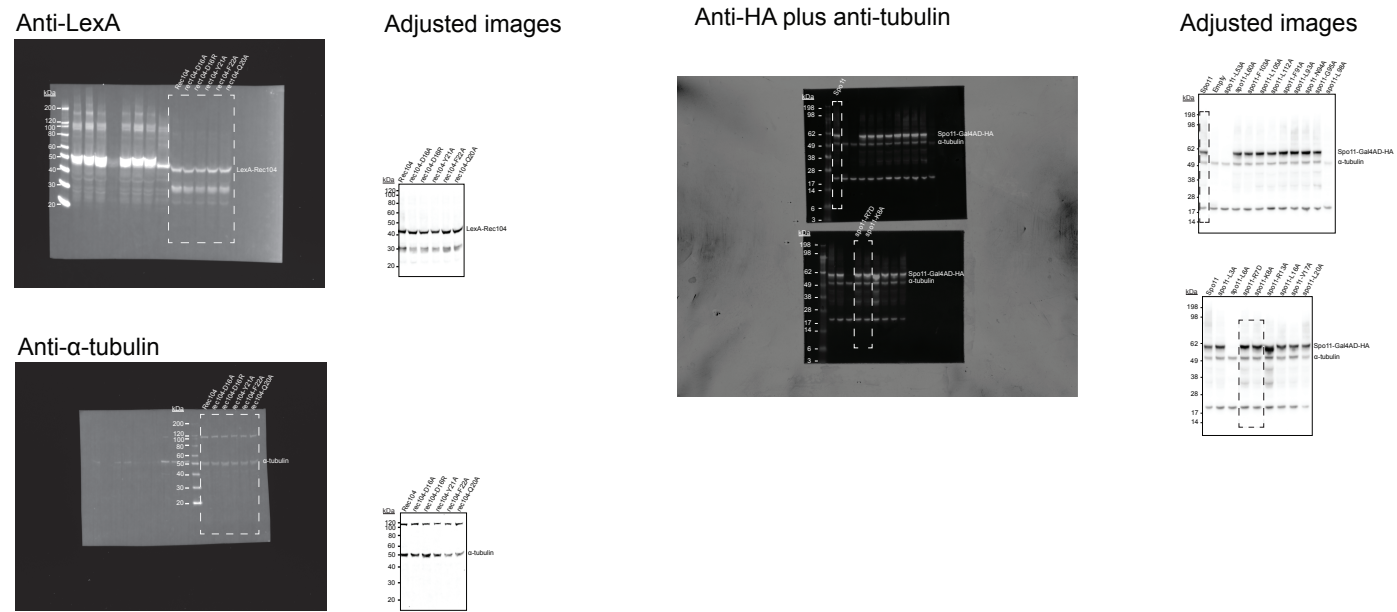

Extended Data Fig. 8 e

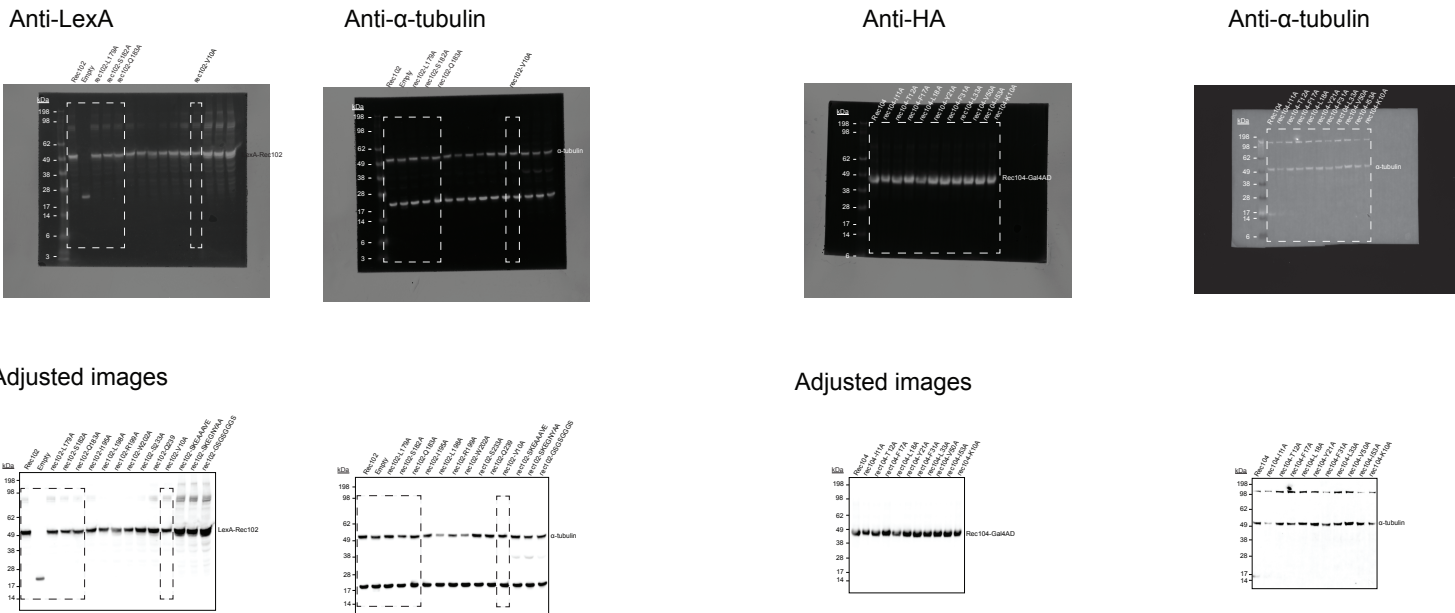

Supplement: Supplementary file 10 — Unprocessed immunoblots. [file 41594_2024_1382_MOESM10_ESM.pdf]
